# Social Media Text Mining Framework for Drug Abuse: Development and Validation Study With an Opioid Crisis Case Analysis

**DOI:** 10.2196/18350

**Published:** 2020-08-13

**Authors:** Tareq Nasralah, Omar El-Gayar, Yong Wang

**Affiliations:** 1 Supply Chain and Information Management Group D’Amore-McKim School of Business Northeastern University Boston, MA United States; 2 College of Business and Information Systems Dakota State University Madiosn, SD United States; 3 The Beacom College of Computer and Cyber Sciences Dakota State University Madiosn, SD United States

**Keywords:** drug abuse, social media, infodemiology, infoveillance, text mining, opioid crisis

## Abstract

**Background:**

Social media are considered promising and viable sources of data for gaining insights into various disease conditions and patients’ attitudes, behaviors, and medications. They can be used to recognize communication and behavioral themes of problematic use of prescription drugs. However, mining and analyzing social media data have challenges and limitations related to topic deduction and data quality. As a result, we need a structured approach to analyze social media content related to drug abuse in a manner that can mitigate the challenges and limitations surrounding the use of such data.

**Objective:**

This study aimed to develop and evaluate a framework for mining and analyzing social media content related to drug abuse. The framework is designed to mitigate challenges and limitations related to topic deduction and data quality in social media data analytics for drug abuse.

**Methods:**

The proposed framework started with defining different terms related to the keywords, categories, and characteristics of the topic of interest. We then used the Crimson Hexagon platform to collect data based on a search query informed by a drug abuse ontology developed using the identified terms. We subsequently preprocessed the data and examined the quality using an evaluation matrix. Finally, a suitable data analysis approach could be used to analyze the collected data.

**Results:**

The framework was evaluated using the opioid epidemic as a drug abuse case analysis. We demonstrated the applicability of the proposed framework to identify public concerns toward the opioid epidemic and the most discussed topics on social media related to opioids. The results from the case analysis showed that the framework could improve the discovery and identification of topics in social media domains characterized by a plethora of highly diverse terms and lack of a commonly available dictionary or language by the community, such as in the case of opioid and drug abuse.

**Conclusions:**

The proposed framework addressed the challenges related to topic detection and data quality. We demonstrated the applicability of the proposed framework to identify the common concerns toward the opioid epidemic and the most discussed topics on social media related to opioids.

## Introduction

### Background

Social media are used by patients to exchange information and discuss different health-related topics [1]. Popular social media platforms, such as Twitter, provide efficient methods of information access for health surveillance and social intelligence [2] and could be used to recognize communication and behavioral themes of problematic use of prescription drugs [3]. Social media have been used in several studies as a resource for monitoring prescription medication abuse [3-7]. The literature shows that clear signals of medication abuse can be drawn from social media posts [5]. Furthermore, the literature used text mining to examine and compare discussion topics to discover the thematic similarity, difference, and membership in online mental health communities [8], provide timely information for epidemiologic surveillance [9], and analyze the public’s reactions to the opioid crisis [10].

### Prior Work

Social media users’ posts are used to better understand providers’ attitudes toward using recovery drugs, such as “naloxone,” to treat opioid addiction [11]. Indeed, social media, such as Twitter, can serve as data sources for approaches that automatically detect opioid addicts and support a better practice of opioid addiction, prevention, and treatment [12]. Several studies have used social media as sources of input data to identify individuals amenable to drug recovery interventions [13] and used text mining to examine and compare discussion topics on social media communities to discover the thematic similarity, difference, and membership in online mental health communities [8].

Kalyanam et al [4] developed a strategy in the field of digital epidemiology to better identify, analyze, and understand trends in the nonmedical use of prescribed medications and drugs through social media by utilizing unsupervised machine learning methods. The results showed that social media data mining could provide insights regarding knowledge from daily life that could support a better practice of opioid addiction prevention and treatment. Cherian et al [6] characterized representations of codeine misuse through analysis of public posts on Instagram using content analysis to identify common themes arising in images. The results showed that codeine misuse was commonly represented with the ingestion of alcohol, cannabis, and benzodiazepines.

Further, Lu et al [7] analyzed Reddit data to gain insight into drug use/misuse by classifying user posts using a binary classifier that predicts transitions from casual drug discussion forums to drug recovery forums. Analysis and results showed that the proposed approach “delineates drugs that are associated with higher rates of transitions from recreational drug discussion to support/recovery discussion, offers insights into modern drug culture, and provides tools with potential applications in combating the opioid crisis” [7]. Jelodar et al [14] examined online discussions to discover knowledge and evaluate patients’ behaviors based on their opinions and discussions about alcohol, using a semantic framework based on a topic model (latent Dirichlet allocation [LDA]) and random forest. The results showed that social media data could be helpful in detecting relevant safety problems in a patient’s daily life.

To demonstrate that the use of machine learning and linguistic rules separately is not enough to achieve better results for information extraction from social media, Jenhania et al [15] proposed a hybrid system combining dictionaries, linguistic patterns, and machine learning to extract structured and salient drug abuse information from health-related tweets. The results showed that the use of a linguistic method based on a dictionary with no dictionary updates is a failed solution. Combining linguistic rules, machine learning, and domain achieved good performance compared with other approaches.

### Goal of This Study

Despite recent advances, there are several limitations that exist when social media data are used for studying drug abuse. First, limitations exist in terms of data relevance and the ability to capture relevant data [6,16]. Second, challenges exist with social media data in terms of completeness and inconsistencies, especially with data collected from multiple resources [17]. Third, user-generated content often includes users’ personal opinions and thoughts, making the task of extracting high-quality information from such data increasingly important [18]. Finally, obtaining high-quality data is a key to avoid any issues in the data preparation step. Several studies reported issues with informal language used on social media [17-19], which could lead to low data quality.

Based on the aforementioned challenges and issues, there is a need to develop a framework that identifies important and relevant quality data on social media to study drug abuse. Furthermore, research that systematically analyzes social media content to study drug abuse in a manner that mitigates challenges and limitations related to topic deduction and data quality is needed.

This research proposed a social media text mining framework for drug abuse that provides a systematic approach to analyze social media data and addresses challenges related to topic deduction and data quality. We demonstrated the applicability of the proposed framework using the opioid epidemic as a drug abuse case analysis by analyzing Twitter data. Twitter was selected because it is an instant day-to-day micro-blogging platform [20] and is widely considered to have an advantage during crises [21]. From a theoretical perspective, this research highlights the importance of developing and adapting text mining techniques for social media data analytics in the context of drug abuse. From a practical perspective, automatically analyzing social media user–generated content can help understand the public themes and topics regarding drug abuse that exist in social media networks.

## Methods

### Social Media Text Mining Framework for Drug Abuse

**Figure 1:**
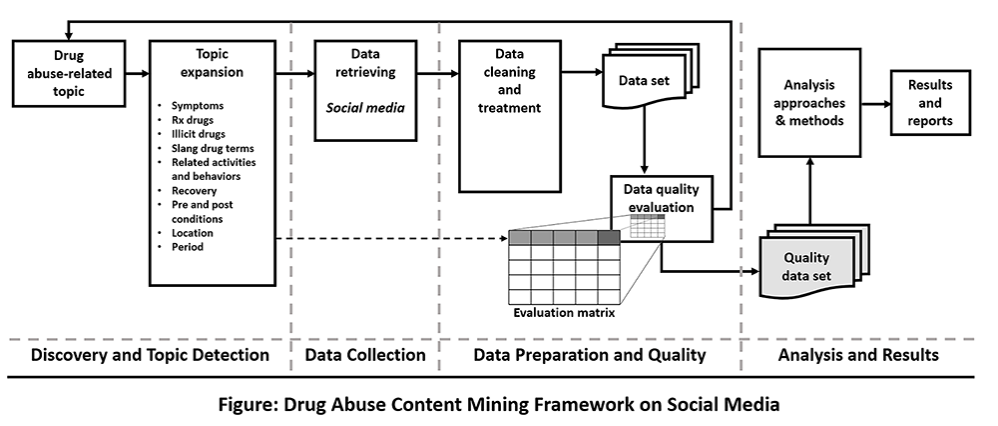
Social media text mining framework for drug abuse. Rx: prescription.

Figure 1 shows the systematic framework to study drug abuse–related topics using social media data. The framework addresses topic detection and data quality challenges. The framework consists of four phases, namely, discovery and topic detection, data collection, data preparation and quality evaluation, and finally, analysis and results.

#### Phase I: Discovery and Topic Detection

**Figure 2:**
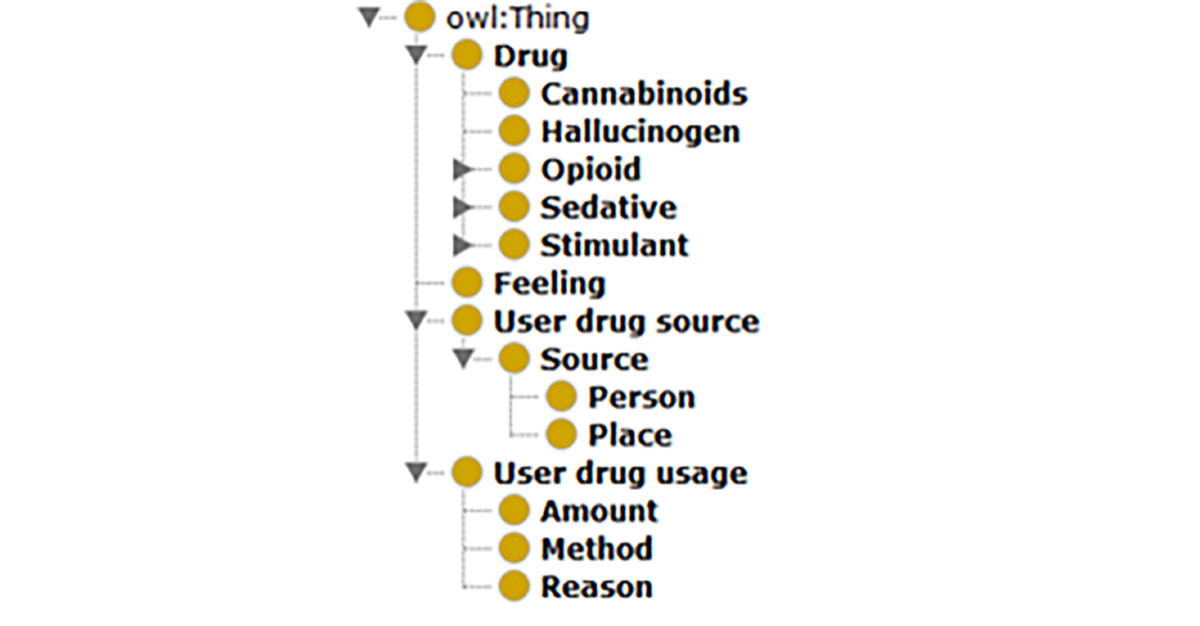
Drug abuse ontology for drug main terms and classes.

According to the literature, challenges and limitations in discovery and topic detection exist [16,17,19]. The interdisciplinary nature of social media data and difficulties in determining the topic that social media posts represent are the most common challenges in social media analysis [17]. To address these challenges, our framework includes a topic expansion step. This step identifies drug abuse–related topics that address the research domain and objectives. To formalize the identification process, we created an ontology for drug abuse based on the literature and expanded the drug abuse ontology proposed by Cameron et al [22] by including related concepts and instances in prescription drug classes (Figure 2) that relate to symptoms, prescription drugs, illicit drugs, slang terms, related activities and behaviors, recovery, conditions, location, and period. The new ontology builds on the ontology by Cameron et al [22] by grouping concepts and instances into themes, reorganizing the concepts hierarchy, and including additional slang keywords and terminologies based on collected data that are related to opioid abuse.

#### Phase II: Data Collection

Data collection is determined by the date range of interest, social media data sources, such as Twitter, relevant keywords to search for posts, and restrictions to impose (language: English, geographic location: United States, etc). The selection of relevant search keywords is based on the proposed ontology in the topic expansion step.

#### Phase III: Data Preparation and Quality

**Figure 3:**
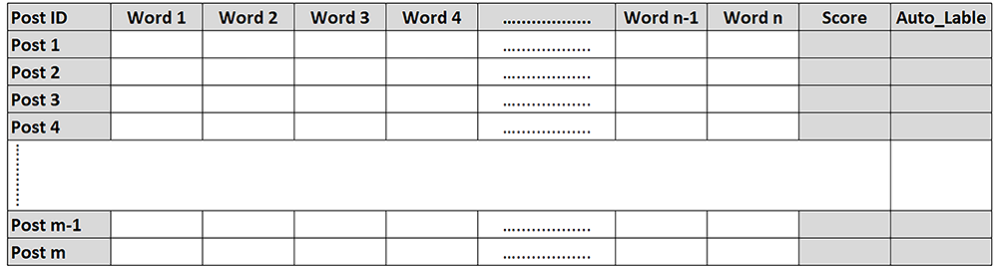
Evaluation matrix for users’ postquality assessment.

The veracity of data leads to issues in data preparation [17]. Therefore, we need to preprocess the collected data and clean it from stop words, punctuations, URLs, etc. To extract quality data for the analysis phase, we evaluated the quality of the data with respect to the terms from the topic expansion step, using an evaluation matrix (Figure 3). The evaluation matrix examines each user’s post in the data set to ensure it includes related terms from the ontology in the topic expansion step.

We automatically generated and populated the evaluation matrix using natural language processing (Python NLTK package) to examine the relevant user tweet. Thereafter, each user’s post (represented as a row) was evaluated against different terms (represented as columns) from the topic expansion step. If the term is present in the post, the value of the term will be one; otherwise, it will be zero.

Evaluation is performed by assigning a data quality score for each post. The value of the quality score was calculated based on the summation of all the term values. The quality score of each user’s post was used as a metric for filtering out low-quality irrelevant posts. Specifically, posts with quality scores from 2 to 10 were retained as relevant posts and those with scores less than 2 or greater than 10 were considered not relevant.

Thresholds were selected based on manual analysis of the collected data. The choice of 2 as the minimum quality score for a post to be relevant was based on the presence of two keywords from the ontology, which increases the possibility of making the post relevant to the topic. The presence of another feature in the post increases the chance of making the context of the post relevant to the study topic. On the other hand, the choice of 10 as the maximum quality score for a post to be relevant was based on manual analysis, where the presence of many words (>10) in a post makes the subject matter and the context of the post too scattered and inaccurate to be relevant to the topic. The evaluation matrix performance was validated against a ground truth. The ground truth represents manually labeled posts.

#### Phase IV: Analysis Approaches and Methods

Once quality data are ascertained, the researcher can choose the suitable data analysis approach based on research questions and objectives. Such approaches could be unsupervised machine learning approaches like topic modeling [23] and supervised machine learning approaches like classification [24].

### Evaluation of the Framework

We instantiated the proposed framework using opioid drug abuse as a case study. To demonstrate and evaluate the proposed topic expansion step in the framework, we instantiated an opioid drug abuse ontology from the proposed drug abuse ontology in Figure 2. To demonstrate the applicability of the opioid drug ontology, we used a sample data set of 10,000 tweets belonging to self-identified opioid users on Twitter. We studied the distribution of the ontology terms and their occurrence over the collected samples. We relied on data collected from Twitter using the Crimson Hexagon platform from June 29, 2018, to April 11, 2019.

To evaluate the performance of the evaluation matrix as the first step in the process, we randomly selected 1000 tweets from the results of the search query. Two independent researchers reviewed the 1000 tweets to determine if each tweet was relevant. We measured the level of agreement using the Cohen kappa interrater reliability metric [25]. Considering the search query as our “base” classifier (classifier 1), where all 1000 tweets were predicted as relevant, we evaluated its performance against the “ground truth” obtained from manually evaluating the relevance of the 1000 randomly selected tweets, using standard data mining and machine learning performance metrics [26]. Using the same 1000 tweets, we applied the evaluation matrix (referred to as classifier 2) to classify the tweets as relevant or nonrelevant based on their quality score. Using the ground truth data obtained earlier, we evaluated the performance of classifier 2 and compared its performance metrics against those obtained for classifier 1.

Since our interest was to identify the different topics that exist in Twitter data about the opioid epidemic, we applied unsupervised text modeling using LDA [23] to extract the different topics that Twitter users have in their tweets. In our case analysis of studying the opioid epidemic, we followed the best practices suggested by Arun et al [27] and computed the term frequency-inverse document frequency (TF-IDF) and perplexity of a held-out test set to evaluate LDA models using a different number of topics. We trained several LDA models with a different number of topics (k) and evaluated the perplexity of a held-out test set. We held out 20% of the data for test purposes and trained the models on the remaining 80%.

## Results

**Figure 4:**
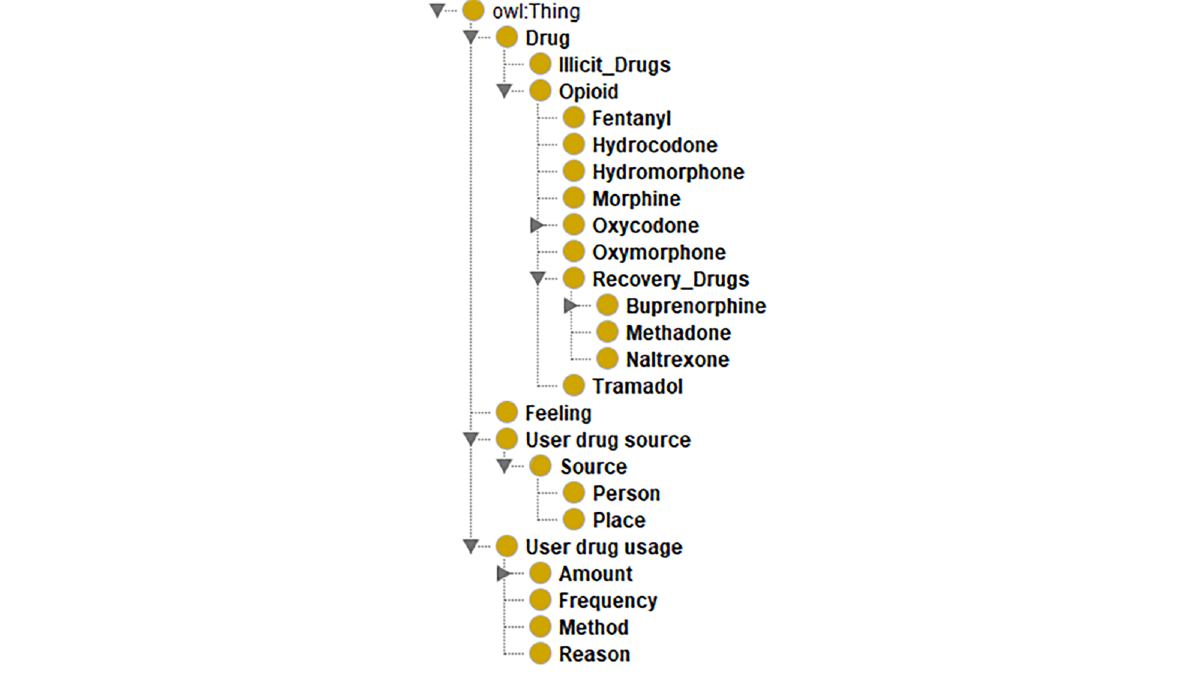
Opioid drug abuse ontology that includes opioid-related terms and concepts.

**Figure 5:**
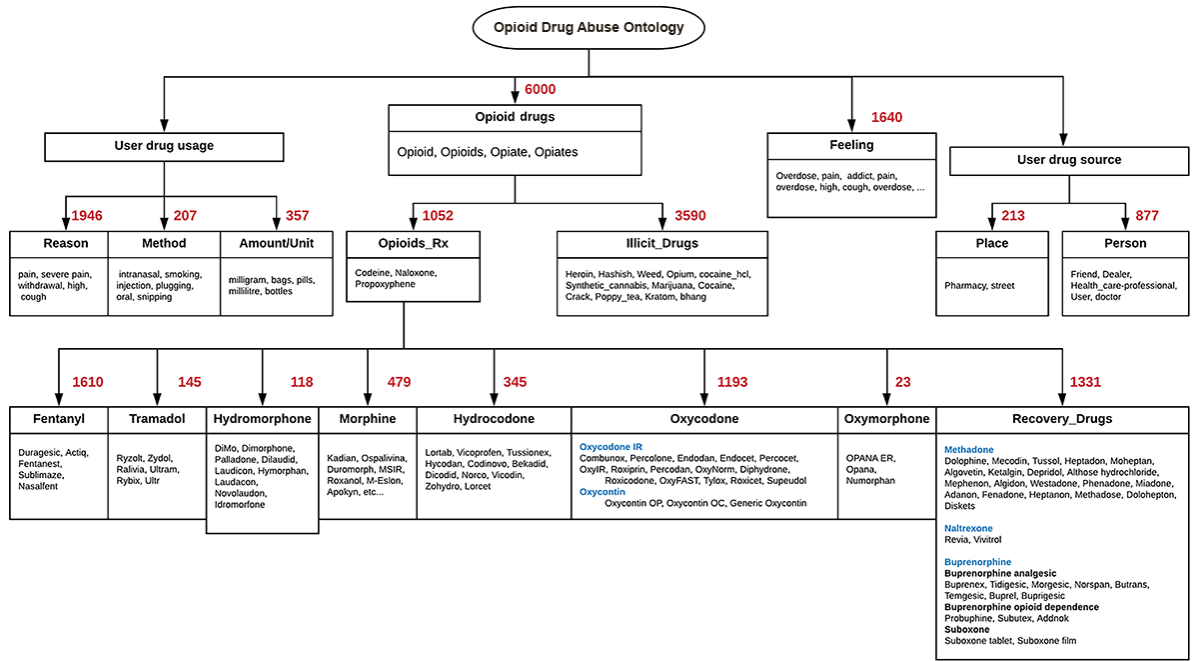
Opioid drug abuse ontology tree hierarchy that reflects the distribution of sample tweets over the ontology concepts and terms. Rx: prescription.

The results demonstrate the instantiation of the four phases of the proposed framework. Regarding phase I, Figure 4 depicts the results of an instantiation of the ontology in Figure 2 for opioid drug abuse, while Figure 5 shows the opioid drug abuse ontology classes using a tree representation. The same post can belong to more than one class in the tree. The number next to each branch in the tree represents the number of tweets based on the node terms. For example, searching relevant tweets using opioid drug–related terms, such as opioid, opioids, opiate, and opiates, yielded 6000 related tweets out of the 10,000 tweets. However, searching tweets using more specific and focused terms that belong to all subclasses of the “opioid drug” class yielded 9886 related tweets. A sample of the defined terms in the ontology can be found in Multimedia Appendix 1.

**Figure 6:**
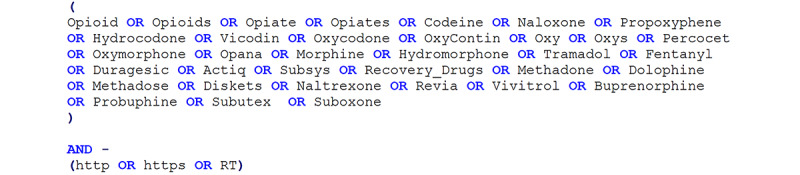
A search query based on the terms and concepts from the opioid drug abuse ontology to collect opioid-related users’ posts.

**Figure 7:**
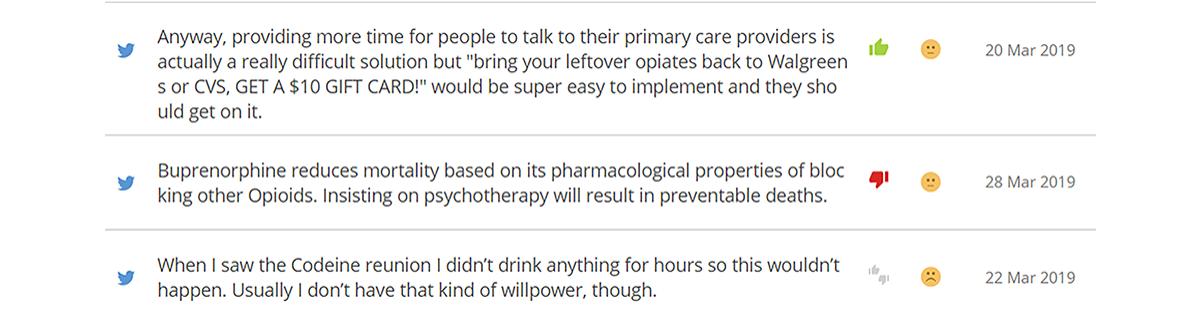
Sample of the collected tweets.

In phase II, using the Crimson Hexagon platform, a social media analytics tool for data collection and analysis, we created a search query (Figure 6) using terms extracted from the opioid drug abuse ontology to retrieve data with no retweets or URLs. For the time period from June 29, 2018, to April 11, 2019, we collected Twitter data related to the opioid epidemic from social media users living in the United States, including practitioners, leaders, patients, journalists, etc, who tweeted about opioids. Overall, we were able to collect 502,830 English-language tweets. Figure 7 shows a sample of the collected tweets.

Reviewing the 1000 randomly selected tweets as part of phase III resulted in 764 relevant tweets and 236 nonrelevant tweets representing the ground truth of the evaluation process. The resultant level of agreement using the Cohen kappa interrater reliability metric was 0.70, which represents moderate agreement [25].

The “base” classifier (classifier 1) (reflecting the search query without the application of the evaluation matrix) defaults to all 1000 tweets predicted as relevant, and this effectively indicates that 764 tweets were categorized as true positive and the remaining 236 tweets were categorized as false positive. The resultant performance metrics for our “base” classifier are summarized using the evaluation metrics under classifier 1 in Table 1.

Using the evaluation matrix (referred to as classifier 2) to classify the 764 relevant tweets representing the ground truth, classifier 2 resulted in 738 tweets classified as relevant (true positives) and 26 tweets classified as nonrelevant (false negatives). From the 236 nonrelevant tweets, classifier 2 classified 190 tweets as nonrelevant (true negative) and 46 tweets as relevant (false positive). The performance metrics using the evaluation matrix are summarized under classifier 2 in Table 1.

The results from Table 1 demonstrate that the proposed evaluation matrix outperforms the manual process. Such results are considered sufficient to adopt the evaluation matrix for evaluating the quality of the collected tweets.

**Figure 8:**
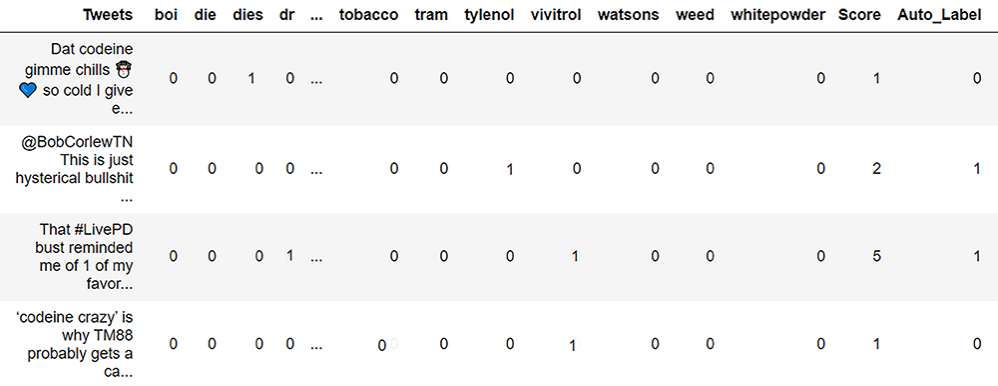
Sample outcomes from the evaluation matrix, where tweets autolabeled 0 are irrelevant and tweets autolabeled 1 are relevant.

We demonstrate the use of the evaluation matrix to automatically evaluate the relevance of the opioid-related tweets. Using the opioid drug abuse ontology, we ended up with more than 250 related terms. To obtain good data quality, we used a variety of opioid drug abuse terms in the evaluation matrix as features. The terms were adapted from the opioid ontology. Figure 8 shows a sample of the evaluation matrix results.

Based on the evaluation matrix score that represents the summation of the occurrence of ontology terms in a tweet, the matrix labeled relevant tweets with 1 if the tweet score was ∈ [2, 10] and nonrelevant tweets with zero if the tweet score was ∉ [2, 10].

According to the evaluation matrix, 366,736 tweets out of the 502,830 collected tweets were deemed relevant (good quality) based on their scores. Multimedia Appendix 1 includes tables showing samples of good quality and excluded tweets. After we obtained the good quality tweet data set, we applied several preprocessing steps to prepare the data for the analysis phase, including the removal of emojis, lemmatization, and tokenization.

**Table 1:** Metrics comparing the performance of manual analysis (classifier 1) and the evaluation matrix (classifier 2) [26].

Variable	Without evaluation matrix (classifier 1)	With evaluation matrix (classifier 2)
Precision	0.764	0.941
Recall	1.000	0.966
F-measure	0.866	0.953
Accuracy	0.764	0.928

**Figure 9:**
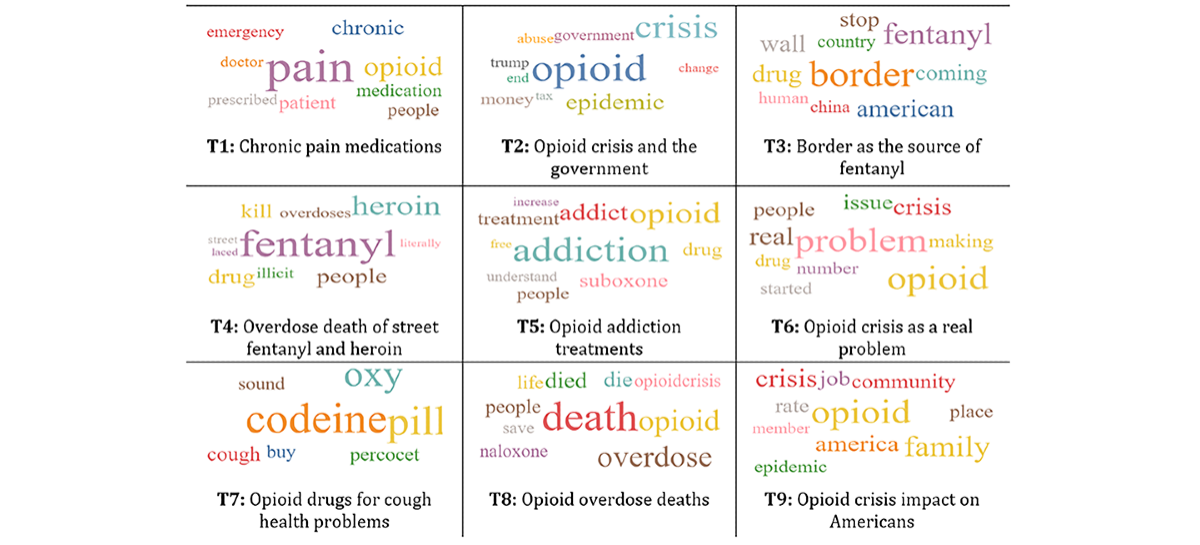
Top nine topic word clouds.

Once we were able to finalize the set of relevant tweets, in phase IV, we applied the LDA topic mining algorithm as noted in the methodology. In that regard, the results showed that the perplexity decreased with an increase in the number of topics but tended to converge at a specific point. This occurred at around 50 topics; hence, we set the number of topics to 50. We examined the LDA model results and manually labelled and grouped 18 topics from among the 50 topics of the public opioid tweets. The topics were labelled by two researchers independently and then reviewed iteratively. Figure 9 shows the word clouds for the top nine topics, with the size of the word representing the unigram TF-IDF score.

For each tweet, the LDA algorithm calculated the probability that tweet *x* belongs to topic *y*. Thereafter, we computed the topics’ weights by determining how many tweets belonged to a specific topic. Table 2 shows the distribution of public opioid tweets over the topics. The most prevalent topics were related to the opioid crisis. Many posts were related to topics, such as chronic pain medications, the opioid crisis and how the US government deals with it, opioids drugs coming across the US border, deaths because of overdose due to opioid fentanyl and heroin, opioid treatments, opioid crisis as a real problem, taking opioid medications for health problems such cough, opioid overdose deaths, opioid crisis impact on American communities, and patients suffering from opioid addiction.

**Table 2:** Public opioid topic weights.

Topic	Description	Top 10 topic words	Topic weight^a^ (N=264,522)
1	Chronic pain medications	Pain, opioid, chronic, med, patient, medication, people, emergency, doctor, and prescribed	40,437 (15.29%)
2	Opioid crisis and government	Opioid, crisis, epidemic, money, government, abuse, trump, end, tax, and change	28,210 (10.66%)
3	Border as the source of fentanyl	Border, fentanyl, drug, wall, American, coming, stop, country, human, and China	25,380 (9.59%)
4	Overdose death from street fentanyl and heroin	Fentanyl, heroin, people, drug, kill, illicit, overdoses, street, literally, and laced	25,226 (9.54%)
5	Opioid addiction treatments	Addiction, opioid, addict, suboxone, drug, treatment, people, understand, free, and increase	18,160 (6.87%)
6	Opioid crisis as a real problem	Problem, opioid, real, crisis, issue, people, making, number, drug, and started	13,966 (5.28%)
7	Opioid drugs for cough and other health problems	Codeine, pill, oxy, shit, cough, percocet, sex, yall, buy, and sound	13,950 (5.27%)
8	Opioid overdose deaths	Death, overdose, opioid, died, die, people, life, naloxone, opioid crisis, and save	13,179 (4.98%)
9	Opioid crisis impact on Americans	Opioid, family, crisis, America, job, rate, community, place, epidemic, and member	11,214 (4.24%)
10	Patient suffering from opioid prescriptions	Patient, doctor, opioid, cancer, prescribing, control, doc, suffering, opioids, and suicide	10,909 (4.12%)
11	Taking opioids after surgery or hospitalization	Day, morphine, feel, surgery, hospital, gave, time, home, needed, and sick	10,326 (3.90%)
12	Illegal market for getting prescription drugs	Drug, prescription, illegal, street, market, dealer, law, opioid, supply, and sell	9948 (3.76%)
13	Legalizing medical marijuana and cannabis	Medical, marijuana, opioid, cannabis, legal, research, pot, study, state, and cannabidiol	9129 (3.45%)
14	People dying from opioids	People, opioid, white, dying, news, crime, crack, folk, black, and house	9012 (3.41%)
15	Public health and substance	Care, health, opioid, substance, public, worse, world, guy, mental, and vote	8065 (3.05%)
16	Opioid addiction and withdrawal	Addicted, opiate, percocet, week, people, opioid, withdrawal, hooked, thinking, and common	7662 (2.90%)
17	Methadone clinic solutions for addiction	High, methadone, solution, fix, clinic, level, crazy, gone, heroine, and wait	4912 (1.86%)
18	School health care education programs	Today, program, school, access, healthcare, policy, jail, recovery, act, and education	4837 (1.83%)

^a^The number represents the number of tweets in each topic, and the percentage represents the proportion of tweets with respect to all tweets.

## Discussion

### Principal Findings

As shown in Table 1, use of the data quality evaluation matrix resulted in a distinct improvement, as depicted by the improvements in accuracy, precision, and F-measure. The slight decline in recall is an artifact of the metric, where all posts generated using the search query were predicted as relevant. The F-measure, which captures precision and recall, depicted a 10% increase in the classification relevance when the evaluation matrix was used.

With respect to the case study, the topics identified using the LDA topic modeling algorithm match the exiting topics in the literature, such as efforts by the former president of the United States to address the opioid epidemic, promotion and legalization of marijuana as an effective alternative for managing pain, marijuana as an alternative to opioids, roles of foreign countries in the epidemic and production of synthetic opioids, advertisements promoting opioid recovery programs [10], and opioids as “medications” to alleviate pain [28].

With respect to medication and pain management, topics 1, 7, 10, and 11 captured the public’s concerns about the prescriptions of chronic pain opioid medications for emergency health conditions and surgery-related chronic pain management. The strategy to address such issues is to change the policies for prescribing chronic pain medications. Example policies and strategies include promoting the responsible use of opioids, reducing the supply of opioids, implementing drug take-back programs [29], tracking and monitoring prescription drug abuse, and reporting electronic prescriptions [30].

With respect to prescription drug abuse, topics 4, 8, 12, and 14 dominated discussions about overdose deaths from taking opioids and illicit drugs, such as heroin, offered by street dealers and other illegal venues. Tightening monitoring and control over such venues (particularly online) can play a major role in the availability of such drugs and can ultimately reduce fatal cases of drug overdose.

With respect to the opioid drug crisis in the United States, topics 2, 3, 6, and 9 reflected discussions focusing on the opioid use crisis, how opioid drugs coming across the US border exacerbate the crisis, and the US government’s actions toward this crisis. Interventions to solve such a situation can involve supporting existing agencies such as customs and law enforcement units and drug interdiction agencies. The results are consistent with findings from the study by Glowacki et al [10], where many discussion topics were related to efforts from the former president of the United States to address the opioid epidemic, warnings from the Food and Drug Administration about mixing opioids with sedatives, and attempts from opioid makers to stop the legalization of marijuana.

With respect to compulsive drug seeking and use treatments, topics 5, 17, and 18 relate to opioid addiction treatments and resources that provide such treatments. Providing individuals and clinics with information about opioid treatment programs and increasing the number of providers of such programs can help in mitigating opioid addiction and overdose problems. Furthermore, providing schools with health care education programs about opioid addiction and recovery programs can create awareness in the community.

With respect to drug legalization for medical or recreational use, topic 13 involved discussions to legalize marijuana for medical and recreational use instead of using opioid prescriptions. Such discussions are in agreement with findings and recommendations regarding the promotion and legalization of marijuana as an effective alternative for managing pain [10,28], as well as advertisements promoting opioid recovery programs [10,31].

Our analysis identified specific discussions that were not identified by prior research. These discussions were mainly about the public’s awareness of problems with opioid overdose, its causes, and its consequences; the benefits of rehabilitation clinics as solutions for opioid addiction and overdose; and the need for health care educational programs at schools.

Overall, the most discussed topics in the analysis can help in understanding the different concerns that the public has around the opioid crisis in the United States. This can serve as a key input for defining and implementing innovative solutions and strategies to address the opioid epidemic.

### Conclusions

Online social media are rich sources of data on an individual’s daily activities and lifestyle. Applying text mining techniques can help in understanding the concerns of online social communities. This study aimed to formulate a systematic analysis approach to obtain good quality social media data sets of drug abuse. We developed a social media text mining framework for drug abuse. We addressed how the framework can help in solving associated challenges related to topic detection and data quality. Further, we demonstrated the applicability of our proposed framework to identify the common concerns toward the opioid epidemic, and we addressed the most discussed topics on social media related to opioids. The insights from the daily posts of public and opioid-addicted social media network users can help provide better opioid prevention, treatment, and recovery strategies. From an information systems perspective, the framework and associated processes can be applied to other domains where there are challenges associated with topic identification and data quality. This research strengthens public health data reporting and collection through social media. With regard to the broader impact of the research results, we expect better insights into drug abuse epidemics.

#### Theoretical and Practical Implications

From a theoretical perspective, this research highlights the importance of developing and adapting text mining techniques to social media data analytics for drug abuse. A particular significance is the emphasis on developing methods for improving the discovery and identification of topics in social media domains characterized by a plethora of highly diverse terms and lack of a commonly available dictionary or language by the community, such as in the case of opioid and drug abuse. The framework addresses problems associated with data quality in such contexts and can be applied to other domains where there are challenges associated with topic identification and data quality.

From a practical perspective, automatically analyzing social media users’ posts can help decision-makers to understand the public themes and topics that exist in online communities. Addressing the most discussed topics on social media related to drug abuse, such as the opioid epidemic, can help understand the problem dimensions and create proper strategies. Moreover, classifying the online social activities of people who are addicted or have been addicted to opioids can help understand the nature of their issues of misusing or overdosing opioid prescriptions, as well as understand user experience. This can help in identifying their concerns and their common issues. Furthermore, it can help in understanding different themes, such as the ways that lead individuals to be addicted, the illicit ways that they obtain opioids, the management of their addiction (if they do manage it), the kinds of medications they use to recover, the other drugs they use or are addicted to, and the types of opioids they are addicted to and their percentages.

#### Limitations and Future Research

Additional refinement of the data quality evaluation matrix is needed. For the study case analysis, additional refinement of the defined categories can further support the applicability of the framework. Finally, there is a need to supplement the collected data with surveys of opioid users to better understand their specific concerns and experiences.

Future research aims to explore the proposed framework using different social media platforms to discover the relations between “opioid” online communities and other online health communities, such as “chronic pain,” “posttraumatic stress disorder,” and “anxiety.” Such communities could have a strong relation with people addicted to opioids and could further improve the effectiveness of detecting drug abuse topics from users’ posts.

## Appendix

Multimedia Appendix 1 Samples of the opioid drug abuse terms and tweets.

## Abbreviations

LDA: latent Dirichlet allocation
TF-IDF: term frequency-inverse document frequency
