# Supplementary material for: Social Media Text Mining Framework for Drug Abuse: Development and Validation Study With an Opioid Crisis Case Analysis
Source: J Med Internet Res. 2020 Aug 13;22(8):e18350. doi: 10.2196/18350 (PMC7446758; doi:10.2196/18350)
Supplement: Multimedia Appendix 1 [file jmir_v22i8e18350_app1.doc]

## Multimedia Appendix

### Sample defined opioid drug abuse terms

| Terms | | Related slang terms |
| --- | --- | --- |
| **Prescription (Rx) Opioids drugs** | |  |
|  | Hydrocodone | Hydrocodones, Hydro, Watsons, etc. |
|  | Codeine | Codiene, Tylenol |
|  | OxyContin | Oxy, Oxys, Oxies, etc. |
|  | Fentanyl | fent, fentanol, fentora, fentanyl, etc. |
|  | Morphine | Morph, Morphy, Morf, Morphie, etc. |
| **Illicit drugs** | |  |
|  | Heroin | ‘white powder’, raw, diesel, H, etc. |
|  | Cannabis | Bud, weed, mg, herb, Hashish, pot, etc. |
|  | Cocaine | Crack, cocain,etc. |
| **Reason for taking the drug** | |  |
|  | Self-medication, pain, severe pain, withdrawal, high, cough, etc. |  |

### Sample good quality tweets

| Tweet | Label |
| --- | --- |
| The opioid crisis is sorta proof that Trump is not an anomaly. It is so stupid that we have become hooked on these drugs at the hands of "infallible" medical science. It shows we are willing to believe anything that sounds like an easier way out than the drudgery of reality | 1 |
| We love you. Removing you from your own show and promoting overdosing on opioids...unreal. Sad. | 1 |
| Oh you don't have to tell me. You know what the pain medication with the least side effects and highest effectiveness for me is? Morphine. ?? I live in a state with a fentanyl epidemic and doctors will not rx it so I am stuck on drugs that make me sick instead. RIP | 1 |
| It's not a lie that the NRA is just like Big Pharma, who are pushing Opioids on Americans, and causing the Opioid crisis. The NRA are "drug" pushers, causing a deadly epidemic of gun violence in America. Yes. They are child killers. | 1 |

### Sample excluded tweets

| Tweet | Label |
| --- | --- |
| Why’s my dad upstairs listening to codeine dreaming .... | 0 |
| "My name is Vic, short for Vicodin" | 0 |
| Codeine crazy is a cult classic | 0 |
| Song: ??percocet molly Me: this is an absolute slapper, not just some run of the mill bop | 0 |
| I put oxy clean with bleach in as fabric softener. I’m losing my mind RIP to my clothes | 0 |
| Sticking to my plan of becoming a Vicodin soccer mom when I grow up | 0 |
